# A Scheme for Enhancing Precision in 3-Dimensional Positioning for Non-Contact Measurement Systems Based on Laser Triangulation

**DOI:** 10.3390/s18020504

**Published:** 2018-02-07

**Authors:** Yassine Selami, Wei Tao, Qiang Gao, Hongwei Yang, Hui Zhao

**Affiliations:** Department of Instrument and Engineering, Shanghai Jiao Tong University, Shanghai 200240, China; selami@sjtu.edu.cn (Y.S.); gaoqiang123@sjtu.edu.cn (Q.G.); yanghongwei@sjtu.edu.cn (H.Y.); huizhao@sjtu.edu.cn (H.Z.)

**Keywords:** laser triangulation, subpixel location, curve fitting, calibration

## Abstract

Laser triangulation allows non-contact measurement in the third dimension. Due to the nonlinearities presented in camera and laser sensor, large range distances are quite difficult to measure with high precision. In order to enhance the precision and accuracy of large range measurement based on laser triangulation, we propose a novel scheme composed of four laser emitters, curve fitting subpixel location algorithm for laser center detection, and the linear regression approach based on the Gaussian model for calibration. When an object performs a 100 mm displacement from a closer to a farther point, our system achieved a repeatability up to ±7 µm, an estimated standard deviation of fitting error within 0.0027 mm, an expanded uncertainty of repeatability within 0.13 mm, an average error variation of rotational plane within 0.15 degree and a nonlinearity error within ±0.04% in full scale. Compared to published results, our proposed method shows an enhancement in accuracy. The error is significantly reduced and maintains at the low level for large ranges, which makes this system applicable and suitable for industrial and indoor applications.

## 1. Introduction

Non-contact measurements play a vital and inevitable role in the modern life. There are different types of measurement systems available; for example, the time of flight (TOF) method presented by Claudio et al. [1] is an ultrasonic distance measurement method. It estimates the time of a traveling and reflecting of an ultrasonic wave from the object to calculate the distance. A similar technique was presented by Palojarvi et al. [2], where a laser pulse was used for signal emission then they use the departure and arrival time of the signal to calculate the distance. Buerkle and Fatikow [3] presented a distance measurement technique based on triangulation principle. Their technique works by emitting a laser beam and calculates the laser point disparity of the object to get the distance; it is a kind of image processing technique and with a simple mechanism. Muljowidodo et al. [4], presented a different scheme based on laser triangulation for an unmanned underwater vehicle, also this technique is based on image processing. Rameshwar [5] presented a piecewise linear calibration method in his paper for height measurement based on laser triangulation, the method allowed successful measurement for large ranges. However, the techniques presented and used for distance measurement in these papers are considered to suffer from a lack of accuracy and cannot be suitable for such applications where high accuracy is required.

In this paper, we adopt laser triangulation principle [6] to achieve large range measurement with high accuracy. The scheme composed of a monocular vision and four laser emitters. The principle of laser triangulation has been used to measure the elevation or depth [7,8] on a surface. The laser source beam projected on a surface has a certain rayon when observed with a camera [9,10,11,12]. In laser triangulation, the laser spot projected on a surface behaves as the reference [13]. The angle created between the camera, reference and the laser allows distance measurement using triangulation principle. In order to enhance the accuracy of the laser triangulation method, the center detection of the laser spot on the object must be accurate. We adopt a sub-pixel positioning curve fitting algorithm of centroid to acquire the sub-pixel center positioning coordinates of the laser spot. First, the centroid method is used for obtaining the sub-pixel location, finally the curve fitting method is used for precise positioning. To overcome limited ranging caused by the depth of focus for a lens and lens distortion, the linear regression based on Gaussian model is proposed for calibration, this calibration method allows measurements for large ranges with high accuracy. The paper is divided as follows. In Section 2, we present the proposed scheme and explain distance measurement flow of our system citing two advantages, Section 3 contains the explanation of the laser spot center detection using the subpixel curve fitting of centroid. In Section 4, we explain the reason for choosing the linear regression based on Gaussian model to calibrate our system. Finally, results are discussed and conclusions are deducted.

## 2. Measurement Principle

### 2.1. Distance Measurement Based on Displacement

**Figure 1:**
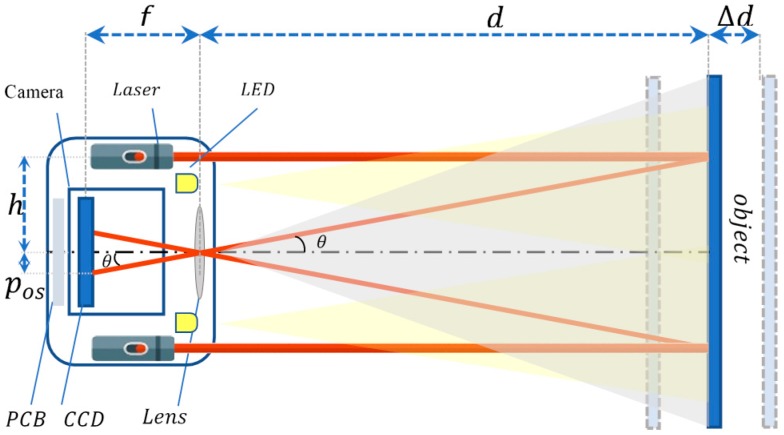
Laser triangulation geometry principle for distance determination.

Figure 1 shows the diagrammatic sketch of the proposed scheme. The system is a square box composed of four main elements: A charge-coupled device (CCD) images capture sensor as an input element, four laser emitters with punctiform emission, four LEDs for illumination to test the functionality and robustness of the system in dark and light environments, and a control board (PCB) to control the system.

The plane of the camera, the laser emitters and the object are parallel. The principle of the system is described as follows. The laser emitter emits a laser beam on the object. The diffused light reflected by the object is collected by the lens and imaging a spot on the CCD. Considering that the laser beam projected on the object is parallel to the center of the principal axis of the camera, the distance can be calculated using triangulation principle. The projection is fixed, thus, the object distance d can be obtained by:

(1) $$d=\frac{h}{\tan\left(\theta \right)}$$

where *d* is the distance between the object and the camera, Δd represents the distance displacement, h is the distance between the camera and the laser emitter, f is the focal length of camera lens, $p_{os}$ is the number of pixels representing the measured difference (also referred as a pixel offset).

**Figure 2:**
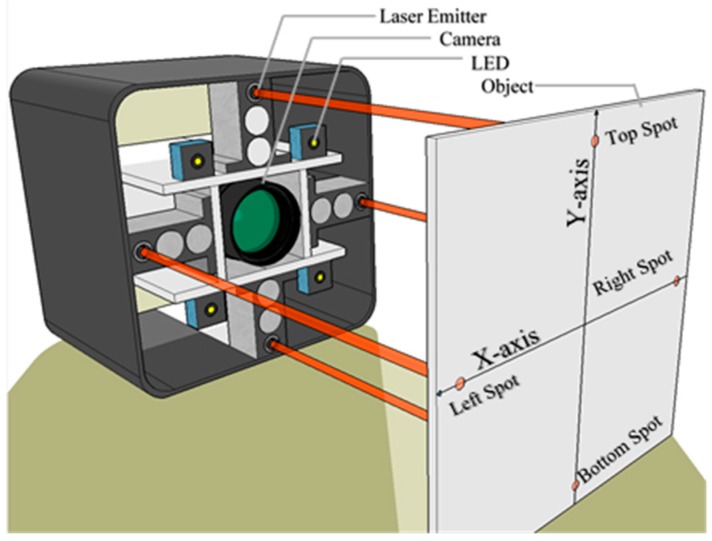
Three-dimensional design of the system.

The system is composed of 4 laser emitters as shown in Figure 2. Hence, 4 values of the distance d are extracted. The laser emitters are directed and distributed symmetrically in horizontal and vertical directions in a region near the center. When the object moves from a near point to a farther point, the top and bottom laser spots on the image perform a displacement along the *Y*-axis only, while the left and the right laser spots on the *X*-axis only. Therefore, the $p_{os}$ coordinates on the *Y*-axis are used to obtain the distance of the top and bottom laser emitters and $p_{os}$ coordinates on *X*-axis are used to obtain the distance from the left and right laser emitters.

**Figure 3:**
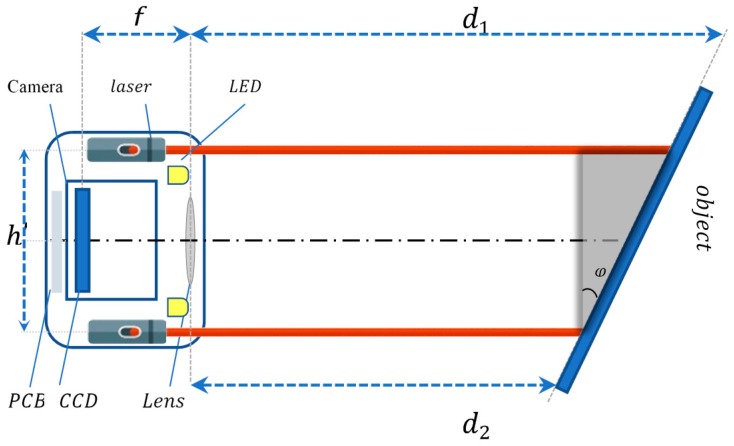
Yaw ($\varphi_{\mathrm{yaw}})$ and pitch ($\varphi_{\mathrm{pitch}})$ angle determination.

Two advantages are obtained from the structure of the proposed scheme, the first advantage is when the object’s plane is parallel to the camera and the laser’s planes, four values of the distance will be obtained, these four values are similar with a slight variation. After using the calibration for known measured distances, the inaccuracies of each laser emitter can be identified. Hence, the error variation can be corrected using various of methods. The second advantage is determination of the yaw ($\varphi_{yaw}$) and pitch ($\varphi_{pitch}$) angle. As shown in Figure 3, the angle φ represents the deviation of the object, forming an inclination with certain degrees. In order to calculate the degree of inclination, the triangulation method is used for this purpose.

(2) $$\varphi =\mathrm{atan}\left(\frac{d_{1}-d_{2}}{h^{\prime }}\right)$$

where $d_{1}$ and $d_{2}$ represent two different distances calculated when the object is on a certain angle, $h^{\prime }$ is the distance between the two laser emitters. The yaw angle ($\varphi =\varphi_{yaw}$) can be determined by the top and bottom laser emitters, and the pitch angle ($\varphi =\varphi_{pitch}$) by the left and right laser emitters.

### 2.2. Laser Spot Center Determination

**Figure 4:**
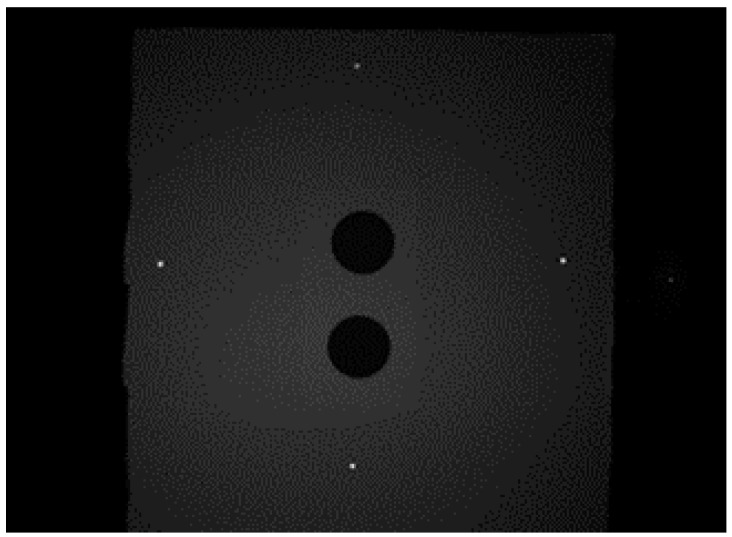
Image of the object and projected laser spots taken from system’s the camera.

As shown in Figure 4, the system’s input is a raw monochromatic image, the plane on the back of the image is the object, and the four laser spots on the object’s plane represent the projection of the laser beam. The preprocessing algorithm performs two main tasks. The first task is adjusting brightness and color intensity to prevent camera saturation, to preserve the roundness of the laser spot projected on the object in order to locate the center accurately. The second task is applying a filter to remove undersized white pixel compared to the size of the projected laser spots. The image is divided into four main regions of interest (ROI), each region is perfectly determined according to each laser’s workspace in order to avoid conflicts during measurements. This procedure is essential to accelerate the calculation rate.

To ensure high accuracy in measurement, precise laser spot positioning on the CCD is required. Traditional algorithms that locates center positioning based on pixel level are not suitable due to the inability to meet the requirements of precise localization in measurement system. Therefore, to enhance the locating precision of the spot center, the sub-pixel location algorithm is adopted. Some of the existing sub-pixel location positioning algorithms such as the centroid method [14], the curve fitting method [15], the correlation method [16] and the moment method [17]. The most common sub-pixel algorithm of center positioning for sub-pixel positioning is the centroid method. The accuracy of the centroid method reaches 0.2~0.5 pixels. However, the previously mentioned methods can be easily affected by the environment, and the positioning error is critically affected by the image signal to noise ratio. In This paper, the sub-pixel curve fitting algorithm of centroid is adopted [18]. The curve fitting technique is considered to be a mathematical method for image processing, its locating accuracy has a direct relation to the specific distribution with the image. First, the centroid technique (3) is used to reach the sub-pixel location, finally the curve fitting is applied for precise locating.

(3) $$\{\begin{array}{l} x_{0}=\frac{m_{10}}{m_{00}}=\frac{\sum_{\left(i,j\right)\in S}iI\left(i,j\right)}{\sum_{\left(i,j\right)\in S}I\left(i,j\right)}=\frac{\sum_{\left(i,j\right)\in S}i}{N}\\ y_{0}=\frac{m_{01}}{m_{00}}=\frac{\sum_{\left(i,j\right)\in S}jI\left(i,j\right)}{\sum_{\left(i,j\right)\in S}I\left(i,j\right)}=\frac{\sum_{\left(i,j\right)\in S}j}{N} \end{array}$$

where ($x_{0}$, $y_{0}$) represent the geometric center of the laser spot in the 2D binary image I(x,y).

- Curve fitting

The laser spot obtained by CCD may be described by the Gaussian distribution, its expression is:

(4) $$y=\frac{1}{\sqrt{2\pi \sigma }}\exp\left[\frac{-{\left(x-\mu \right)}^{2}}{2\sigma^{2}}\right]$$

where μ is the mean or expectation of the distribution, σ is the standard deviation, $\sigma^{2}$ is the variance.

For easier integration into the logarithm Equation (4) is written in the following form:

(5) $$\ln y=-\frac{{\left(x-\mu \right)}^{2}}{2\sigma^{2}}+\ln\frac{1}{\sqrt{2\pi \sigma }}$$

Equation (5) is a quadratic curve on x, hence, a parabola fitting to target is sufficient, which simplified the calculations.

- Curve fitting of centroid

The centroid method is used to find center positioning of the image in this paper, followed by the curve fitting method of the vertical and the horizontal direction positioning on the pixel point. For the horizontal direction, according to Equation (5), the form of quadratic curve can be set as:

(6) $$y=Ax^{2}+Bx+C$$

According to the expression of the grayscale’s value of an image, the output’s grayscale value of each pixel is acquired by:

(7) $$f_{nx}=\overset{n+\frac{1}{2}}{\underset{n-\frac{1}{2}}{\int }}\left(Ax^{2}+Bx+C\right)dx$$

Assuming that the coordinate solved by centroid method is $(x_{0}$, $y_{0})$, and this pixel’s grayscale value is $f_{0x}$, set the order number of the point as 0. According to Equation (7) we have:

(8) $$f_{0x}=\overset{\frac{1}{2}}{\underset{-\frac{1}{2}}{\int }}\left(Ax^{2}+Bx+C\right)dx=\frac{A+12C}{12}$$

Centering on the pixel point, then take a pixel point backward and forward respectively, to get two points $f_{-1x}$ and $f_{1x}$. According to Equation (7) we have:

(9) $$f_{-1x}=\overset{-1+\frac{1}{2}}{\underset{-1-\frac{1}{2}}{\int }}\left(Ax^{2}+Bx+C\right)dx=\frac{13A+12B+12C}{12}$$

(10) $$f_{1x}=\overset{1+\frac{1}{2}}{\underset{1-\frac{1}{2}}{\int }}\left(Ax^{2}+Bx+C\right)dx=\frac{13A-12B+12C}{12}$$

Solving simultaneously Equation (8)–(10), we can get the constants A, B and C:

(11) $$A=\frac{\left(f_{-1x}+f_{1x}-2f_{0x}\right)}{2}$$

(12) $$B=\frac{\left(f_{-1x}-f_{1x}\right)}{2}$$

(13) $$C=\frac{26f_{0x}-f_{-1x}-f_{1x}}{24}$$

The apex of the quadratic curve can be solved, as shown in Equation (14).

(14) $$x=\frac{f_{-1x}-f_{1x}}{2\left(2f_{0x}-f_{1x}-f_{-1x}\right)}$$

The calculation procedure of the vertical direction is the same as that of the horizontal direction. The logarithm of the grayscale values is taken in Equation (14), and the sub-pixel center in the horizontal direction can be gotten:

(15) $$x_{sub}=x_{0}+\frac{\ln f_{-1x}-\ln f_{1x}}{2\left(2\ln f_{0x}\;-\ln f_{1x}-\ln f_{-1x}\right)}$$

As well, the vertical direction of sub-pixel center can be calculated the same way:

(16) $$y_{sub}=y_{0}+\frac{\ln f_{-1y}-\ln f_{1y}}{2\left(2\ln f_{0y}\;-\ln f_{1y}-\ln f_{-1y}\right)}$$

**Figure 5:**
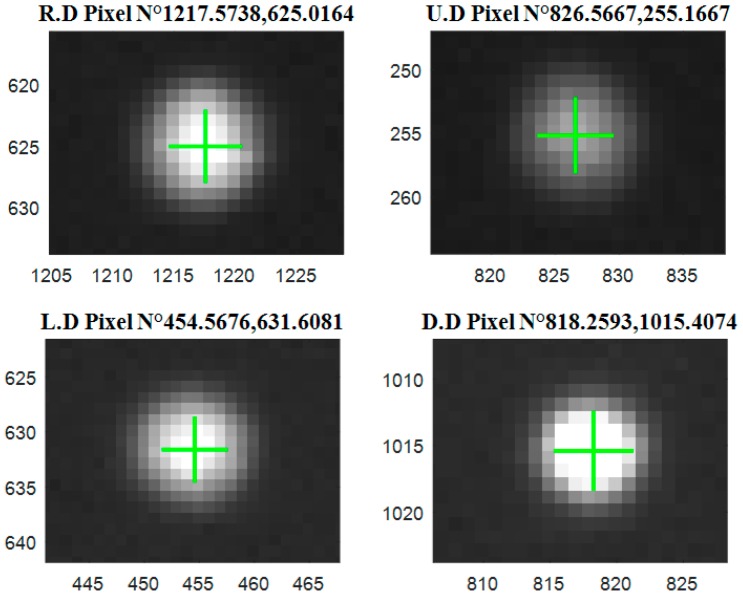
Results of laser’s spot subpixel center positioning.

According to the proposed method, the laser spots center sub-pixel coordinates in Figure 4 are extracted, and the results of the proposed algorithm are shown in Figure 5.

The center location is given in the format X, Y in each picture, the digits after the decimal point are estimates values.

### 2.3. Calibration

Calibration is required before doing distance measurement. Various calibration methods are available [19]. The distance expression Equation (1) shows a directly proportional relationship between the distance displacement (Δd) and the angle θ. In practical applications, this relation cannot be satisfied over large ranges. Moreover, if the system is calibrated for large measuring ranges, it will lose its accuracy, which raises a trade-off between the length of measuring range and the system accuracy [5]. Therefore, the linear regression based on Gaussian fitting model Equation (17) is applied here.

(17) $$\delta =\overset{n}{\underset{i=1}{\sum }}a_{i}exp\left[-{\left(\frac{x-b_{i}}{c_{i}}\right)}^{2}\right],$$

where a is the amplitude, b is the centroid positioning, c represents the peak width and n is the number of peaks to fit.

**Figure 6:**
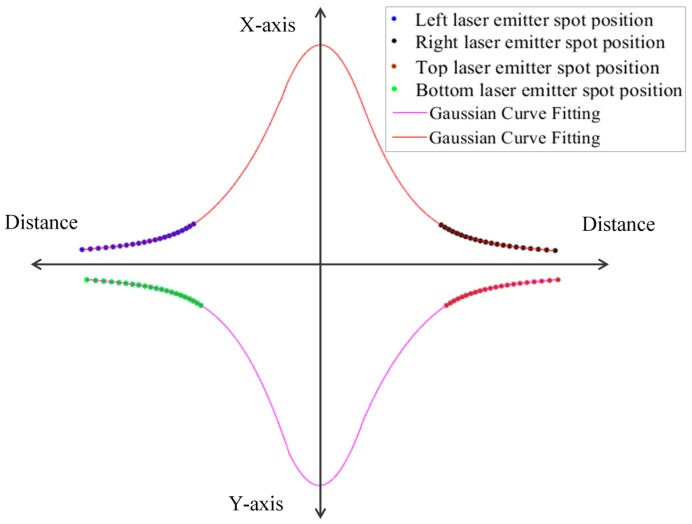
Sketch of fitting laser spots coordinates with the correspondent distance using linear regression curve fitting based on Gaussian distribution model.

In this approach, the calibration is applied on two laser emitters at the same time (Figure 6), the fitting curve takes the bell curve shape. We found that the linear regression based on Gaussian model is the best fit for our system. The Equation (17) is applied on each two symmetrical laser emitters. The Equation (18) is obtained by fitting the left and right laser emitters, where $x_{hl}$ and $x_{hr}$ represent the horizontal coordinates of the laser spots on the CCD of the left laser emitter and right laser emitter, respectively. The Equation (19) is obtained from fitting the top and bottom laser emitters where, $y_{vt}$ and $y_{vb}$ represent the vertical coordinates of laser spots on the CCD of the top laser emitter and bottom laser emitter, respectively. Objects with known distances are taken for calibration. The system is calibrated in each 5 mm for the whole range, considering the range is divided into 5 mm segments.

(18) $$\delta_{h}=a_{1}*exp\left(-{\left(\frac{x_{hl}-b_{1}}{c_{1}}\right)}^{2}\right)+a_{2}*exp\left(-{\left(\frac{x_{hr}-b_{2}}{c_{2}}\right)}^{2}\right)$$

(19) $$\delta_{v}=a_{1}*exp\left(-{\left(\frac{y_{vt}-b_{1}}{c_{1}}\right)}^{2}\right)+a_{2}*exp\left(-{\left(\frac{y_{vb}-b_{2}}{c_{2}}\right)}^{2}\right)$$

The system shows four values of the distance, these values have a small discrepancy, where each laser perform the best for a defined distance segment when the object’s plane is parallel to the system. We used the mean deviation method to increase the accuracy (20).

(20) $$MD=\frac{\sum \left|x_{i}-\mu \right|}{N}$$

where $x_{i}$ is each value, μ is the mean and N is the number of values.

By using this method, we analyzed the error deviation of the four laser emitters when performing a full range measurement, then we extract four coefficients, these coefficients based on minimization of the error deviation on each laser emitter output. Once these coefficients are obtained we multiply them to each laser output. Finally, we obtained one distance of the object with the best accuracy taking account all four laser emitters.

## 3. Experimental Results

### 3.1. Equipment presetting

**Figure 7:**
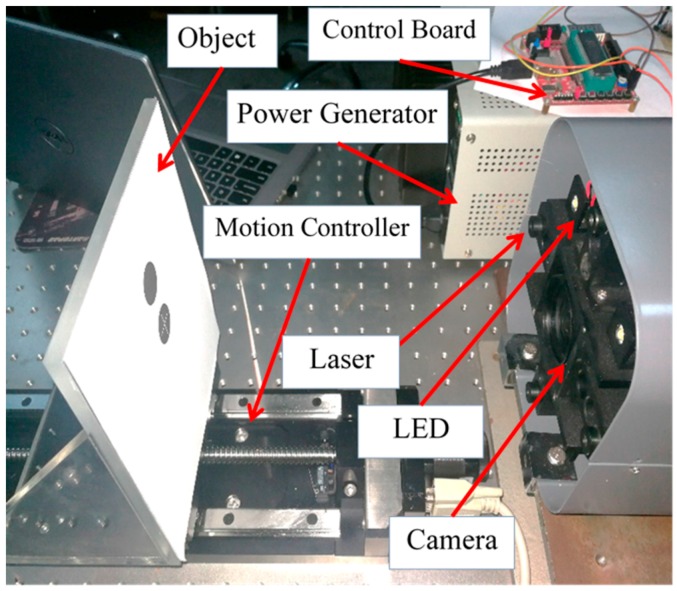
The equipment and devices used to achieve non-contact distance measurement.

The experimental system is designed as in the form of a 15 × 15 cm square box (Figure 7), which consists of the camera, lens, four laser emitters, four LEDs, control board, test targets and moving rail (motion controller), the distance between the laser emitters and camera is 66 mm.

Camera: in this experiment, the MER-200-14GM/GC 200 Mega Pixel GigE Sony CCD Industrial Video Camera (Daheng (Group) Co., Ltd., Beijing, China) was used. The pixel’s number of the camera is 1628 × 1236, the pixel’s size is 4.4 μm, the output flash can be synchronized signal.

Lens: The lens’s reference is LM4NCM (KOWA Company Ltd., Nagoya, Japan) with the focal length of 4.4 mm, image size 1/1.8″, viewing angle of 89.0° × 76.6° × 61.3° (D × H × V) and focus range of 0.1 m ∼ ∞.

Laser: The laser is M650D5-3M-1235 (Xi’an Minghui Optoelectronics Technology Co. Ltd., Xi’an, China) red dot laser module that can be modulated, characterized by the wavelength of 650 nm.

Microcontroller: AT89C52 8-bit microcontroller (Microchip Technology Inc., Chandler, AZ, USA) from Atmel's, has 256 bytes of RAM and a capacity of 8 KB of Flash Programmable and Erasable Read Only Memory (PEROM).

Motion controller: ZXPC130 motion controller (Shanghai Zhengxin Optical Instrument Co. Ltd., Shanghai, China) was used for tracking the rail control, it drives the test target forward and backward, and can provide accurate travel distance value, as the distance calibration and benchmark.

Object: is a rectangular shaped surface, in order to ensure the accuracy of range, it is preferred to use a white surface.

### 3.2. Repeatability Test

**Figure 8:**
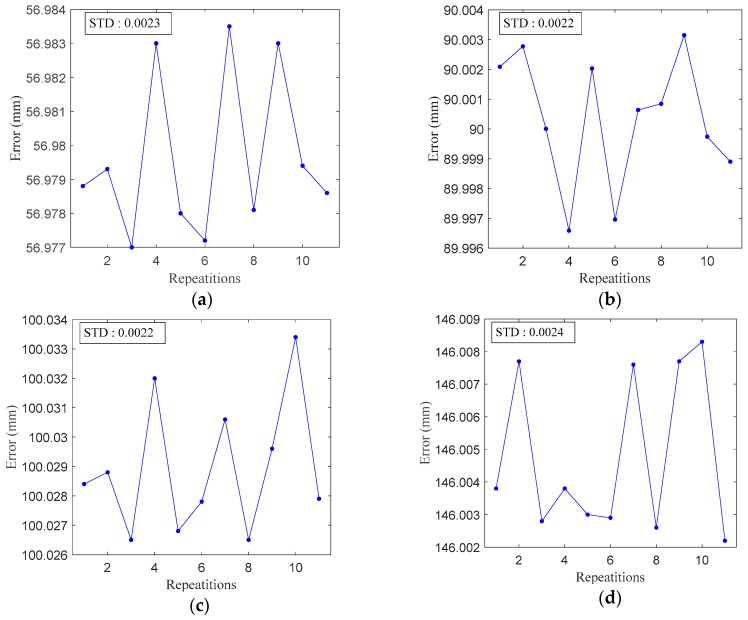
(**a**) Repeatability results on closer point displacement; (**b**) repeatability results on average point displacement; (**c**) repeatability results on average point displacement; (**d**) repeatability results on farther point displacement.

In the experiment, the object performs a displacement from a closer point to a farther one, the displacement is taken at a range of 50 mm to 150 mm. As shown in Figure 8, the repeatability test is taken from four different distance ranges, results show that our system achieved a repeatability of approximately ±7 μm and the STD is within 0.0027 mm. The expanded uncertainty of repeatability measurement is calculated by U=ku(y), where U is the expanded uncertainty of measurement, k is the standard coverage factor and u(y) is the standard uncertainty. The standard coverage is taken *k* = 2. Results show that the expanded uncertainty of repeatability is within 0.13 mm.

### 3.3. Rotational Plane Test

**Figure 9:**
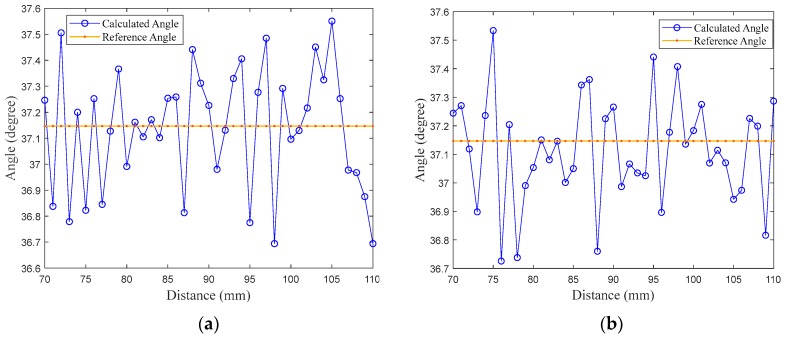

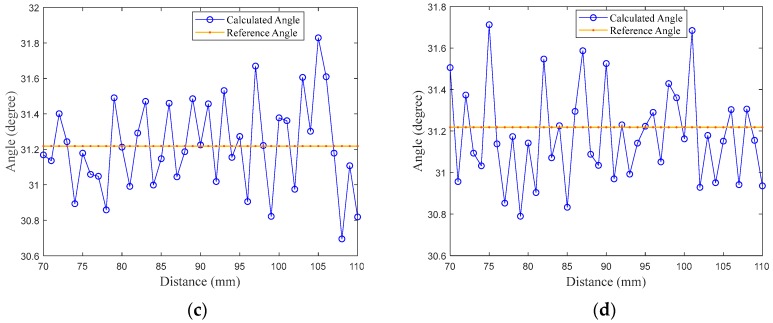
(**a**) Error variation of $\varphi_{yaw}$ angle in measurements; (**b**) error variation of $\varphi_{pitch}$ angle in measurements; (**c**) error variation of $\varphi_{yaw}$ angle in measurements; (**d**) error variation of $\varphi_{pitch}$ angle in measurements.

As shown in Figure 9, the experimental test of the rotational plane took a place where tests were performed on two angles 37.14 degrees and 31.21 degrees at range of 70 mm to 100 mm. Results show that the average error variation of the rotational angle for both $\varphi_{yaw}$ angle and $\varphi_{pitch}$ angle is within 0.15 degree.

### 3.4. Nonlinearity Test

**Figure 10:**
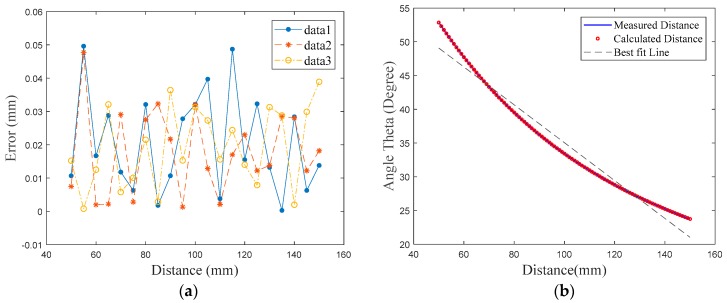
(**a**) Non-linearity result using three data of the calculated distance; (**b**) Non-linearity results compared with the measured distance, calculated distance and best fit line.

The nonlinearity is expressed by $\left(x_{t}-x_{r}\right)/l_{r}$, where $x_{r}$ is the calculated distance, $x_{r}$ is the distance taken from the ZXPC130, also known as the real distance, and the $l_{r}$ is the tested range. In our experiment, the measurements data were obtained by performing object displacement from a closer point to a farther one with an increment of 0.1 mm. As shown in Figure 10, this task was repeated three times. Results show that the nonlinearity between the system and the ZXPC130 is within ±0.04% in Full Scale.

## 4. Conclusions

In this paper, we repost a non-contact measurement method based on laser triangulation along with the experimental implementation. Laser detection and image processing for parameter extraction have been discussed in detail and measurement results are presented. In order to enhance the precision and accuracy of the measurement system based on laser triangulation, it is requisite that the detection of laser spot center must be precise, to meet the requirements for this procedure, the curve fitting subpixel location algorithm of centroid is proposed. This algorithm is applied for spot center positioning precision improvement and to locate the laser spot center down to sub-pixel level. For improving the non-linearity, the linear regression based on Gaussian model approach is applied during calibration. The experimental results reveal that the repeatability has reached ±7 µm, an estimated standard deviation of fitting error within 0.0027 mm, an expanded uncertainty of repeatability within 0.13 mm, an average error variation of rotational plane within 0.15 degree and the nonlinearity error down to ±0.04% in full scale. These results show noticeable improvement compared to the current available methods in terms of absolute error, relative error and standard deviation error, the error maintains at the low value with only a slight variation in the whole range.

Our current design of the range of measurement system based on laser triangulation is still larger than other range measurement systems, which makes it not suitable to be implemented in integrated systems. Nevertheless, the performance of non-contact measurement systems based on laser triangulation has the highest accuracy and has its own application fields.
